# NormaCurve: A SuperCurve-Based Method That Simultaneously Quantifies and Normalizes Reverse Phase Protein Array Data

**DOI:** 10.1371/journal.pone.0038686

**Published:** 2012-06-28

**Authors:** Sylvie Troncale, Aurélie Barbet, Lamine Coulibaly, Emilie Henry, Beilei He, Emmanuel Barillot, Thierry Dubois, Philippe Hupé, Leanne de Koning

**Affiliations:** 1 Institut Curie, Paris, France; 2 Department of Translational Research, Institut Curie, Paris, France; 3 INSERM, U900, Paris, France; 4 Mines ParisTech, Fontainebleau, France; 5 CNRS UMR144, Paris, France; University of South Florida College of Medicine, United States of America

## Abstract

**Motivation:**

Reverse phase protein array (RPPA) is a powerful dot-blot technology that allows studying protein expression levels as well as post-translational modifications in a large number of samples simultaneously. Yet, correct interpretation of RPPA data has remained a major challenge for its broad-scale application and its translation into clinical research. Satisfying quantification tools are available to assess a relative protein expression level from a serial dilution curve. However, appropriate tools allowing the normalization of the data for external sources of variation are currently missing.

**Results:**

Here we propose a new method, called NormaCurve, that allows simultaneous quantification and normalization of RPPA data. For this, we modified the quantification method SuperCurve in order to include normalization for (i) background fluorescence, (ii) variation in the total amount of spotted protein and (iii) spatial bias on the arrays. Using a spike-in design with a purified protein, we test the capacity of different models to properly estimate normalized relative expression levels. The best performing model, NormaCurve, takes into account a negative control array without primary antibody, an array stained with a total protein stain and spatial covariates. We show that this normalization is reproducible and we discuss the number of serial dilutions and the number of replicates that are required to obtain robust data. We thus provide a ready-to-use method for reliable and reproducible normalization of RPPA data, which should facilitate the interpretation and the development of this promising technology.

**Availability:**

The raw data, the scripts and the NormaCurve package are available at the following web site: http://microarrays.curie.fr.

## Introduction

The technology of Reverse phase protein arrays (RPPA) [1], first described in 2001 [2], is a quantitative microformat dotblot approach. It consists in depositing very small amounts of protein extracts onto microscope slides covered with nitrocellulose. Each spot contains 1 ng or less of material and one array can contain up to five thousand spots. Each array is then labeled with an antibody that specifically recognizes a protein of interest. Thus, RPPA is the opposite of forward arrays, also termed antibody arrays [3], where a large selection of antibodies is fixed on the arrays and incubated with one protein extract per array. The advantages of RPPA, compared to antibody arrays, are the small amounts of samples that are required and the possibility to compare protein expression among a large number of samples in the same experiment. As for antibody arrays, the major constraint in RPPA lies in the quality of the used primary antibodies, and systematic validation of their specificity in Western Blot is required. Given the advantages of RPPA, the technology has gained interest notably in the field of cancer proteomics [4], [5]. Examples of successful applications include the identification of activated signaling pathways in different types of cancer [6]–[8] and the identification of prognostic biomarkers [9]–[11]. However, a major issue that is still under development concerns the quantification and the normalization of the data. Indeed, serial dilutions are generally made of each sample, which allows appreciating the dynamic range of an antibody. From these serial dilutions, one relative protein expression level needs to be obtained for each sample for further analysis. This step is termed the quantification of the data. Next, the normalization of the data aims to correct for potential sources of variability that do not reflect biological differences in protein expression between the samples under investigation. These include 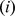 differences in the total amount of protein extracts that were deposited, 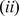 differences in the fluorescence background intensities and 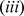 spatial effects on the slides.

Several quantification methods have been proposed. Some of them use a sample-by-sample strategy [12], [13] with linear or logistic models. In this case, a curve is fitted to the serial dilutions for each sample separately, and from this curve the final protein expression level of the sample will be read. Next, these models were improved by applying a joint strategy using all the samples of the array to fit the curve [14]. The joint logistic strategy was shown to improve the accuracy and the dynamic range of the estimated protein expression levels over sample-by-sample estimations [14]. In addition, Hu *et al.* [15] showed that a non-parametric approach is more flexible than the logistic model and may be applied to a greater set of data. Their algorithm is applied array by array and it is implemented in the SuperCurve R package [16]. Besides SuperCurve, a mathematically simpler model, called SerialCurve, was proposed by Zhang *et al.* [17]. Instead of modeling the response curve of an antibody, this model characterizes the relationship between signals in successive dilution steps.

SuperCurve and Serial Curve are currently the most efficient quantification methods. However, they do not normalize the data, *i.e.* they do not remove external sources of variability. Given the high sensitivity (up to the attomol range) and the high precision of RPPA (CV of 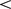
15%), such variations are expected to bias the results [18], [19]. Few publications propose normalization methods: Neeley *et al.* [20] proposed a normalization step, to be applied after SuperCurve, which mainly removes inter-array variability. Another method, called microenvironment normalization, was developed to remove spatial effects within an array and is applied before SuperCurve algorithm [21]. Although very powerful, this method requires many positive control spots and thus significantly diminishes the number of samples that can be analyzed on one array. In conclusion, no satisfying methods for intra-array normalization of RPPA data are currently available.

Here, we propose models to simultaneously quantify and normalize RPPAs. We chose to base our models on SuperCurve quantification, rather than on SerialCurve, since non-parametric models are more flexible and may in some cases better fit observed RPPA data [15], [16]. Four different normalization models were tested. To validate our results, an experiment using a purified protein (Chk2) was used. In this experiment, human samples, mouse samples and known concentrations of Bovine Serum Albumin (BSA) solutions are studied, with or without the addition of exogenous Chk2. The ability of our models to remove spatial effects and to correct for variations in the total amount of spotted proteins is then investigated. All our results are validated by cross-validation and show that the best performing model takes into account three parameters for normalization: one negative control slide, one slide with a total protein stain, and spatial covariates within the array. This model, which we call NormaCurve, allows robust and reproducible normalization of RPPA data.

## Materials and Methods

### Cell lines and Protein Extraction

Cell lines from ATCC have been used in our experiments: NIH-3T3 (ATCC CRL-1658), MCF10A (ATCC CRL-10317), BT20 (ATCC HTB-19) and T47D (ATCC HTB-133) and Jurkat T (ATCC TIB-152). Cell lines are grown in appropriate medium supplemented with 1% penicillin/streptomycin (Invitrogen 15140-122) and 10% foetal calf serum (Invitrogen 10500-064), except when serum starvation is applied. For protein extraction, cells are washed twice in PBS and harvested in hot Laemmli buffer (50 mM Tris pH = 6.8, 2% SDS, 5% glycerol, 2 mM DTT, 2.5 mM EDTA, 2.5 mM EGTA, 1x HALT Phosphatase inhibitor (Perbio 78420), Protease inhibitor cocktail complete MINI EDTA-free (Roche 1836170, 1 tablet/10 mL), 2 mM Na3VO4 and 10 mM NaF). Extracts are boiled for 10 min at 100°C, passed through a fine needle to reduce viscosity and centrifuged 10 min at 15000 rpm. The supernatant is harvested and stored at −80°C. Protein concentration is determined (Pierce BCA reducing agent compatible kit, ref 23252).

### RPPA Experiment

Purified Chk2 protein (Abnova, H00011200-P01) is added to cell extracts or to BSA (Sigma-Aldrich) solutions and detected using a monoclonal antibody against Chk2 (Cell Signaling Technology 3440). The following samples are deposited onto nitrocellulose covered slides (Schott Nexterion NC-C) using a dedicated arrayer (Aushon Biosystems 2470). The design is summarized in the Table 1 and consists of the following extracts:

1. NIH-3T3 cells: the antibody against Chk2 does not recognize the murine protein. Thus, total protein staining is expected to be high while background levels are expected with the anti-Chk2 antibody. The background level corresponds here to non-specific binding and autofluorescence of the nitrocellulose.
2. NIH-3T3 cells + purified Chk2 protein: both total protein staining and anti-Chk2 staining are expected.
3. BSA alone: total protein staining is expected to be high while background levels are expected with the anti-Chk2 antibody.
4. BSA + purified Chk2 protein: both total protein staining and anti-Chk2 staining are expected
5. MCF10A cells: both total protein staining and anti-Chk2 staining are expected,
6. MCF10A cells + purified Chk2 protein: both total protein staining and anti-Chk2 staining are expected
7. Several control cell lines (BT20, jurkat, serum-starved MCF10A, T47D)

For each sample, 15 2-fold serial dilutions are deposited, starting at 1 mg/ml. For the extracts 1 to 4, starting concentrations of 0.8, 0.9, 1, 1.1 and 1.2 mg/ml were used, complemented or not with respectively 0.033, 0.038, 0.042, 0.046 and 0.05 ng/ml purified Chk2. The aim of these varying starting concentrations is to introduce a variability in the spotted amount of total protein, in order to test the ability of our models to correct for this. Samples were divided over two 384-well plates. Each well contained 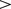
20 µl of extract. In the first well plate, all wells were used and the plate remained open for 125 minutes during printing. In the second well plate, one fourth of the wells was used and the plate remained open for 42 minutes. Total printing time was 3 h 48 min and humidity was kept at 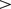
60% during the entire printing process to avoid evaporation. All samples were deposited 6 times on each array (technical replicates). A custom printing was used in order to distribute samples as randomly as possible over the array.

**Table 1:** ModelSC 1: Capacity of the different models to normalize for the varying amounts of total protein spotted.

Array	Sample	pval_φ_	pval*_s_*	pval*_c_*	pval*_cs_*
1	BSA	3.5969e-05	0.1449	0.0420	0.3053
2	BSA	8.2330e-06	0.0991	0.0802	0.2175
3	BSA	1.5165e-06	0.5173	0.0003	0.0063
4	BSA	3.9442e-04	0.3741	0.0016	0.2308
5	BSA	8.2330e-06	0.0991	0.0802	0.2175
1	3T3	3.3674e-08	0.0710	0.0616	0.0644
2	3T3	1.0865e-06	0.2752	0.0561	0.0536
3	3T3	2.1981e-07	0.1603	0.6549	0.2578
4	3T3	1.6289e-10	0.2555	0.1080	0.2672
5	3T3	1.0865e-06	0.2752	0.0561	0.0536

Represented are the p-values of the amount effects without neither 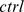 nor 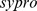 (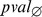), with 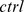 (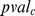), with 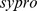 (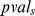), with 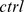 and 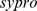 (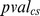).

Five arrays are stained with the total protein stain Sypro Ruby (noted sypro). For this, arrays are incubated 15 min in 7% acetic acid and 10% methanol, rinsed twice in water, incubated 10 min in Sypro Ruby protein blot stain (S11791, Invitrogen) and rinsed again.

In addition, five arrays are labeled with anti-Chk2 antibody (CST 3440) and five arrays are labeled without primary antibody (negative control, noted ctrl), using an Autostainer Plus (Dako). Briefly, slides are incubated with avidin, biotin and peroxydase blocking reagents (Dako) before saturation with TBS containing 0.1% Tween-20 and 5% BSA (TBST-BSA). Slides are then probed overnight at 4°C with primary antibodies (or without primary antibody for negative controls) diluted in TBST-BSA. After washes with TBST, arrays are probed with horseradish peroxidase-coupled secondary antibodies (Jackson ImmunoResearch Laboratories) diluted in TBST-BSA for one hour at room temperature. To amplify the signal, slides are incubated with Bio-Rad Amplification Reagent for 15 minutes at room temperature. The arrays are washed with TBST, probed with Cy5-Streptavidin (Jackson ImmunoResearch Laboratories) diluted in TBST-BSA for one hour at room temperature and washed again in TBST.

The processed slides are dried by centrifugation and scanned using a GenePix 4000B microarray scanner (Molecular Devices). Spot intensity was determined with MicroVigene software (VigeneTech Inc).

### SuperCurve Model and Extensions

#### Initial SuperCurve

Hu *et al.* [15] proposed a non-parametric model to quantify relative protein expression levels from RPPA experiments. The model is 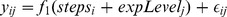 where 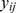 is the intensity measured by the scanner at the 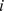
th dilution step for the 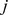
th sample, 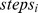 is the dilution step, 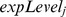 is the median effective relative protein expression level (called EC50), 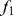 is a non-parametric monotonically increasing function and 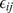 is the random error with a median assumed to be 0. The 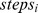 values are log-scaled dilution factors centered on their median (*i.e* equal −2, −1, 0, 1 and 2 for the respective dilution factors 1/16, 1/8, 1/4, 1/2 and 1). This model corresponds to ModelSC1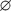 of Table 2. In this model, the function 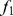 and 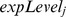 are unknown but estimated using the following iterative algorithm (see [15] for details):

1. for each sample 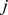, the initial estimation of 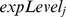 is computed using the following logistic function:
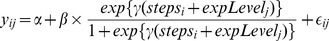
(1)where 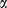, 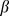, 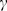 and 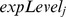 are unknown parameters.

2. from all the samples and based on the initial estimates of 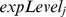, the function *f*_1_ is estimated by a constrained quadratic b-spline via the R package cobs.

3. conditionally on the estimated curve 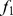, the concentrations 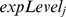 are estimated by a non-linear regression series by series. A dilution series corresponds here to the intensities of a given sample with all its dilution steps.

4. The steps 2 and 3 are iterated twice.

This algorithm is applied array by array and was implemented in the R package SuperCurve [16]. While allowing the quantification of the relative protein expression levels 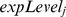 at the step 0, SuperCurve does not take into account potential sources of variability which may bias the measurements. Therefore, we propose extented SuperCurve models in what follows in order to improve the signal-to-noise ratio of the data.

**Table 2:** The initial SuperCurve model (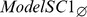) and the extended SuperCurve models.

Name	∅	*c*	*s*	*cs*
ModelSC1	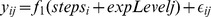	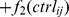	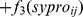	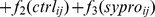
ModelSC2	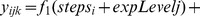	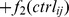	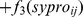	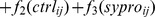
	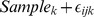			
ModelSC3	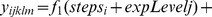	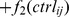	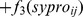	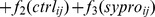
	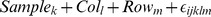			
ModelSC4	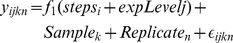	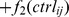	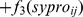	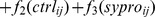
	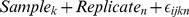			

The best model we propose is bolded.

#### Extended SuperCurve

Based on SuperCurve, we propose models taking into account several covariates which can be separated into two main groups. The first group of covariates corresponds to features which depend on the experimental design. They are the 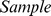 effect which takes into account the differences between the spotted samples, the covariates 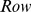 and 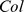 which take into account spatial effects on the array, the 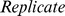 covariate which takes into account the fact that a sample is spotted in at least two replicates within an array. The covariates 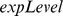 and 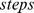 also fall into this group. The second group of covariates corresponds to features which are not directly quantified on the array of interest. They are the covariate ctrl which corresponds to the intensities of the control array without primary antibody and the covariate sypro which corresponds to the intensities of the Sypro Ruby array. These covariates are used to correct the background level and the total amount of spotted proteins, respectively. We defined four models (ModelSC1 to ModelSC4) depending on which covariates from the first group are included (see Table 2). In addition, for each ModelSC, four sub-models are tested depending on which covariates from the second group are considered. The suffixes 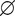, c, s and cs are added to the name of the model in order to distinguish the model with neither ctrl nor sypro, with only ctrl, with only sypro and with the ctrl and sypro, respectively. For instance, the test of the ModelSC2 with neither ctrl nor sypro will be noted ModelSC2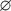 while the test with both ctrl and sypro covariates will be noted ModelSC2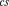. Five ctrl slides and five sypro slides have been performed. From the 25 possible combinations of (ctrl, sypro), nine were tested here. These nine combinations use each array twice.

For all models, a non-parametric function 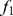 assesses the 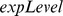 and 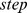 effects as in the initial SuperCurve model. For ctrl and sypro, a linear relationship between the intensities of the ctrl (or sypro) and the intensities of the specific antibody arrays (here anti-Chk2 array) was tested and gave poor results (not shown). Thus, non-parametric functions were used.

In order to add these covariates to the initial SuperCurve model (ModelSC1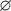), a generalized additive model (gam) of the R package *mgcv* [22] was added. In a gam model, the linear predictor is given by a user specified sum of smooth functions of the covariates plus a conventional parametric component of the linear predictor. The likelihood of gam models is modified by the addition of one or more quadratic penalty coefficient matrices for each smooth function. Each penalty matrix is multiplied by an associated smoothing parameter assessed by the minimization of the REML criterion. The penalities are chosen to minimize an estimator of the resulting mean squared predictor error [22]. Contrarily to the cobs function, the gam function does not take into account monotonicity contraints. Consequently, the use of this function induces the loss of monotonicity in the curve estimate.

The model fitting is performed in the same way as in the initial SuperCurve algorithm except the second step which is modified as follows. Based on the initial estimates of 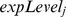, all the parameters of the model (either smooth functions or parametric terms) are estimated by a gam model via the R package *mgcv*.

In ModelSC4, as the 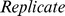 effect is considered as a random effect, the third step of the algorithm detailed above has been modified to assess relative protein expression levels from all the series of a sample instead of series by series like in the other models.

The SuperCurve models of Table 2 are applied array by array and was implemented into the initial SuperCurve package.

### Validation Criteria

In order to compare the different models, we defined three criteria: a cross-validation criterion, a regression criterion and the correlation coefficient. The cross-validation (CV) criterion establishes a model using a training set, which is then validated on a separate set of samples in order to control the robustness and the generalization of a model. The regression criterion reflects how representative the fitted curve is for the real data. The correlation coefficient corresponds to the correlation between the true protein concentrations and the relative expression levels estimated by the model.

**1. Cross-validation (CV) criterion.** For the samples NIH-3T3, BSA and MCF10A, the concentrations of Chk2 are known (either absolutely or up to a delta as there is an unknown base level expression of Chk2). From these samples, both a training set and a test set were built (this procedure is repeated 30 times). Without loss of generality, let us consider the ModelSC1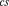. On a given training set and test set, a 5-fold CV criterion is computed as follows:

- On the training set (4/5 of the data): estimates of the parameters of the non-parametric functions 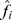 and of the other covariates,

- On the test set (1/5 of the data):

* Estimate of 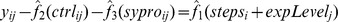,

* From the true concentrations 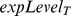, 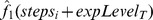 is computed,

* The cross-validation criterion is then:


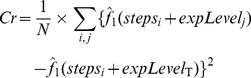
(2)

where corresponds to the total number of spots. This criterion is the most important because it reflects the generalization and the robustness of the model.

**2. Regression criterion.** Corresponds to the REML coefficients of the gam function. We remind that the gam smoothing regression uses a penalization parameter to avoid overfitting.

**3. Correlation coefficient.** Corresponds to the correlation coefficient between the true concentrations and the relative expression level estimated by the ModelSC in the training set.

### Normalization of the Total Amount of Spotted Proteins

As mentioned previously, small variations in the concentrations of total protein are voluntarily introduced in order to assess the ability of our models to correct for these differences. The two samples NIH-3T3 and BSA are thus spotted starting from five varying amounts : 0.8 mg/ml, 0.9 mg/ml, 1.0 mg/ml, 1.1 mg/ml and 1.2 mg/ml. Each starting concentration is then serially diluted 2-fold to obtain 15 dilution steps. The following two step procedure was then used:

1. First, the observed intensities 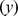 were normalized according to the two control arrays (ctrl and sypro) given the normalized intensities yNorm. Four cases can then be distinguished whatever the ModelSC of Table 2: models without covariate (Equation 3), with only ctrl (Equation 4), with only sypro (Equation 5) and with the two covariates (Equation 6).
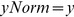
(3)

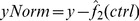
(4)

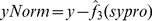
(5)

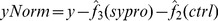
(6)


2. The significance of the effect of the five amounts 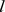 (0.8, 0.9, 1.0, 1.1, 1.2 mg/ml) is tested on the samples BSA and NIH-3T3 via the following linear model 7 where 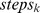 corresponds to the dilution step, 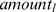 corresponds to the amount effect and 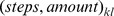 corresponds to the interaction between the two effects, 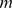 is the sample and 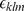 the normal residuals:
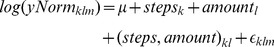
(7)


### Spatial Effect Evaluation

To check if our normalization ModelSC removes spatial effects, the significance of the two effects, 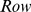 and 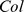, was tested before normalization on the observed intensities (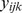) and after normalization on the estimated residuals (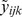) of the ModelSC of Table 2. This test was performed on any ModelSC, even if they do not contain the spatial covariates 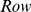 and 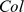 (ModelSC3) in order to check if the other tested covariates (negative control and total protein stain) can remove spatial effects or not. Equation 8 is the linear model taking into account the effect of the 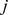
th row and of the 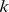
th column for the 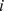
th sample. The significance of the covariates 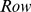 and 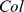 is tested by Fisher tests.
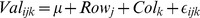
(8)where



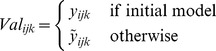



### Required Number of Replicates

We addressed how many technical replicates are required to evidence a significant difference between two samples S1 and S2. Each sample is composed of 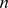 replicates. We want to know the minimum difference between the mean relative protein expression of two samples (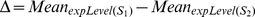) required to be significant. This test can be written as follows: 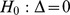 against the hypothesis 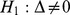. Two parameters may vary: 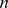 the number of replicates within each sample and 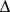 the difference between the two mean expression levels after applying SuperCurve. By varying these two parameters, the power of the test can be calculated. The power corresponds to the probability of rejecting the null hypothesis 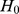 while 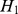 is true.

To perform such a power analysis, the estimation of the intra-array variability is required. Indeed, the higher the intra-array variability is, the higher the mean difference 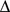 must be in order to have a relevant power (a relevant power is usually fixed from 80%). The intra-array variability (*i.e* the variance of the residuals 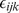) is assessed by the linear mixed-effect model 9 [23] taking into account the sample fixed-effect (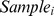) and the array random-effect (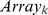) whose variance 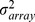 corresponds to the inter-array variability.
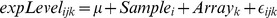
(9)


## Results

We aim to present and validate new models allowing normalization of RPPA data for possible sources of variability that do not represent differences in the expression levels of the protein of interest. These include fluorescence background signal, differences in the total amount of deposited protein and spatial bias on the arrays. All statistical models were described in the Material and Method section.

**Figure 1:**
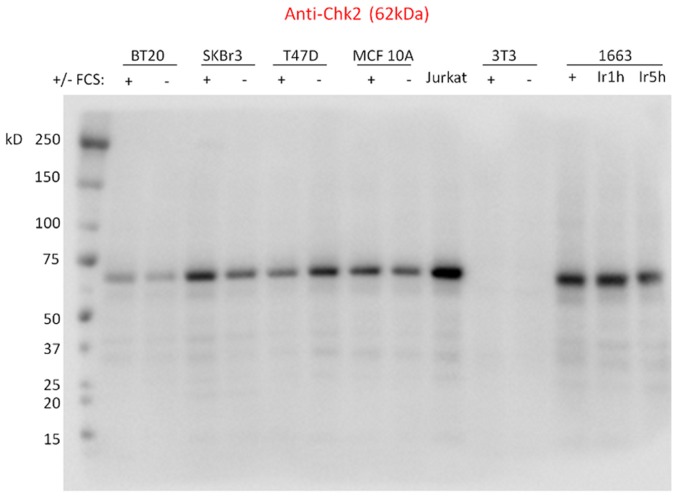
Western Blot. Western Blot analysis of Chk2 protein levels on a panel of different cell lines shows a single band at the expected size and no signal in the mouse cell line NIH-3T3. Molecular weights (kD) are indicated next to the protein ladder (first lane). FCS: Fetal Calf serum, Ir: irradiated.

In order to design an RPPA experiment with known concentrations of protein, we used purified Chk2 protein. Chk2 is a medium-sized protein (around 60 kDa) involved in cell cycle arrest and DNA damage response. Our antibody against Chk2 proved highly specific in western blot analysis on human samples and does not recognize the murine protein (Figure 1). Thus, exogenous human Chk2 can be added to mouse cell extracts (NIH-3T3) resulting in known Chk2 protein concentrations, within the physiological context of a cell lysate. In addition, we added recombinant Chk2 to human cell extracts (MCF10A) and to solutions of Bovine Serum Albumin (BSA). We choose not to deposit the recombinant protein alone, since this does not represent a normal situation of antibody binding and detection. The design of the RPPA arrays is detailed in the Materials and Methods.

**Figure 2:**
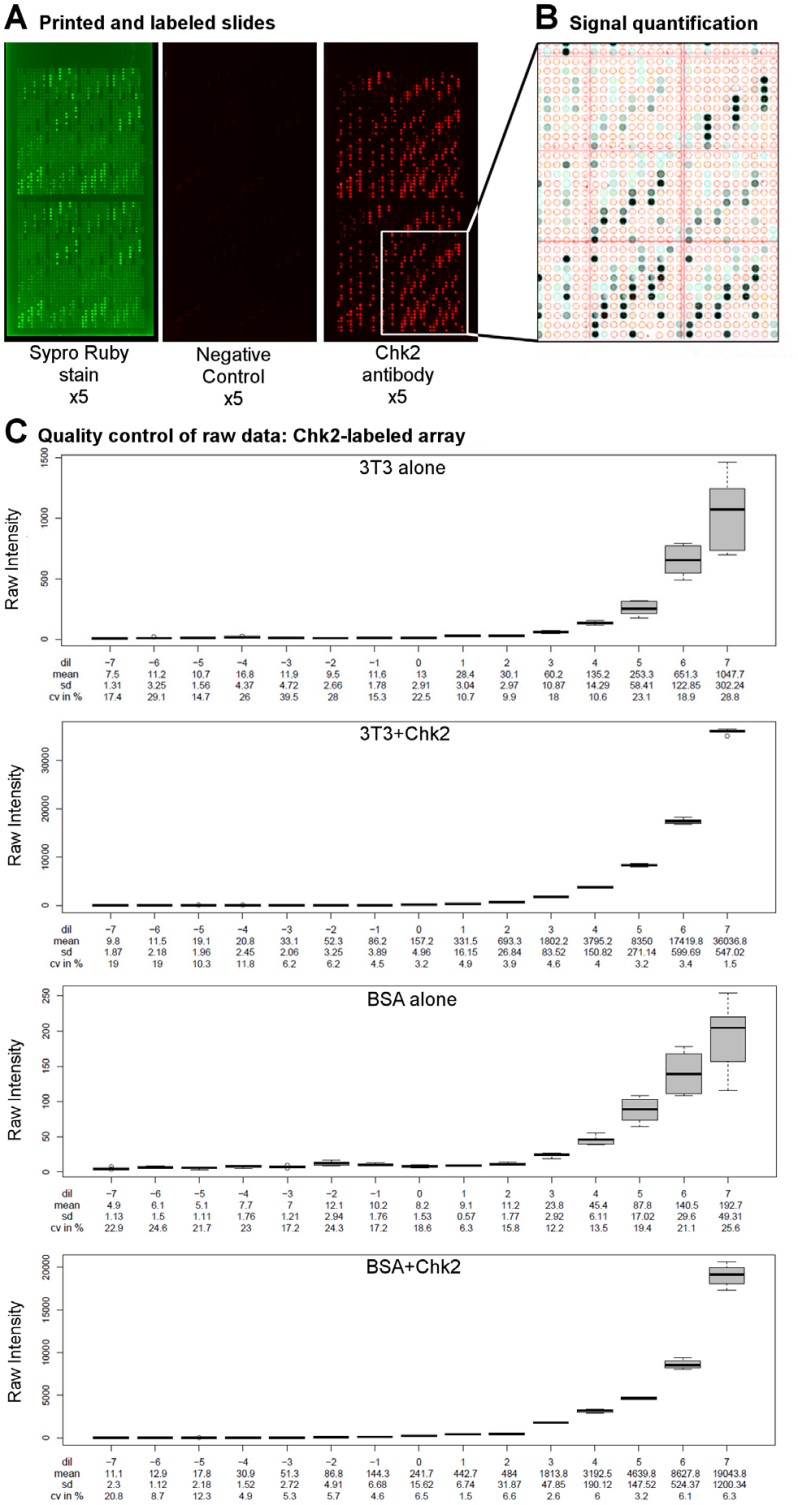
Quality control of raw RPPA data. A. Representative images of the Sypro Ruby labeled slides (left), the negative control slides (middle) and the anti-Chk2 labeled slides (right). No spatial bias was detected visually. On each array, two identical blocks (superarrays) have been printed. The upper block of 40×40 dots is thus a replicate of the lower block. Within each block, samples are deposited in three replicates. Thus, in total, there are 6 replicates per array. B. Dot detection and quantification using MicroVigene software allows to convert images into quantitative numbers and gives rise to raw data. C. Raw data obtained on a representative Chk2-labeled array are plotted against the 15 serial dilutions for the indicated samples. Dilutions (dil) are centered around 0 and indicated below each graph. Mean, standard deviation (sd) and the coefficient of variation (cv) are indicated for each dilution below each graph. Please note the differences in scaling between the four graphs.

Three different stainings were applied: five arrays were labeled with anti-Chk2 antibody, five arrays were labeled without primary antibody (negative control, noted ctrl), and five arrays were stained with the total protein stain Sypro Ruby (noted sypro). None of the slides showed visible spatial bias and the negative control slides showed low background levels (Figure 2A). Relative intensities of each spot were determined using MicroVigene software (Figure 2B) and quality control of the raw data was performed. Boxplots, representing the raw intensities of 6 technical replicates for each dilution step, demonstrate that replicates are highly reproducible (Figure 2C; please note the scaling differences). To further demonstrate the reproducibility of our replicates, we calculated the coefficient of variation (CV, defined as (Standard Deviation/Mean Intensity) ×100) for each sample and each dilution step, on each array (Figure 2C). Median CV of all arrays, samples and dilutions steps was 13.40%. However, we noticed that low intensities are associated with high CVs and vice versa (Figure S1). Indeed, median CV for samples with near-background intensities (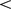
1000) was 16.5%, while median CV for samples with intermediate intensities (between 1000 and 10,000) was 9.6%, and median CV for samples with high intensities (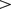
10,000) was 3.9%. Observing high CVs on low intensities is not surprising, since small variations on very low intensities give rise to high CVs. For example, a sample for which the intensity of the replicates ranged from 32 to 102, the mean intensity was 66.1 and the CV 42.6%. Since these values remain within the background noise, a high CV for low intensities is not problematic. In addition, we ensured that good correlations exist between the 5 replicate slides. Indeed, mean Pearsons correlation coefficient was 0.98 for Chk2-labeled slides, 0.72 for negative control slides and 0.99 for Sypro Ruby stained slides. In conclusion, the quality of our raw data is satisfactory in all aspects and we can thus pursue with data analysis.

To start with, we compared parametric and non-parametric models to analyze these data. In agreement with Hu et al., 2007, we observe that non-parametric data better fit the data, in particular for the negative control slide (not shown). We therefore chose the non-parametric model SuperCurve as the basis for our development. The different normalization models that we developed, presented in the Material and Methods, have been applied on these raw data. The evaluation of the normalization described below (cross-validation estimation of relative protein levels, spatial effect and correction for total amount of protein and for spatial effects) uses only one set of ctrl and sypro arrays. The reproducibility of the results with others sets of ctrl and sypro arrays is studied afterwards.

### Estimation of Relative Protein Expression Levels

We use three validation criteria (cross-validation, regression and correlation criteria), described in the Material and Methods, to assess how well the different normalization models (Table 2) predict protein expression levels. The cross-validation (CV) criterion establishes a model using a subset of the samples, called the training set, and is then validated on a separate set of samples in order to control the robustness and the generalization of a model. The regression criterion reflects how representative the fitted curve is for the real data. The correlation coefficient corresponds to the correlation between the true protein concentrations and the relative expression levels estimated by the model. Better prediction of the protein expression levels result in a lower cross-validation criterion, a lower regression criterion and a higher correlation coefficient.

**Figure 3:**
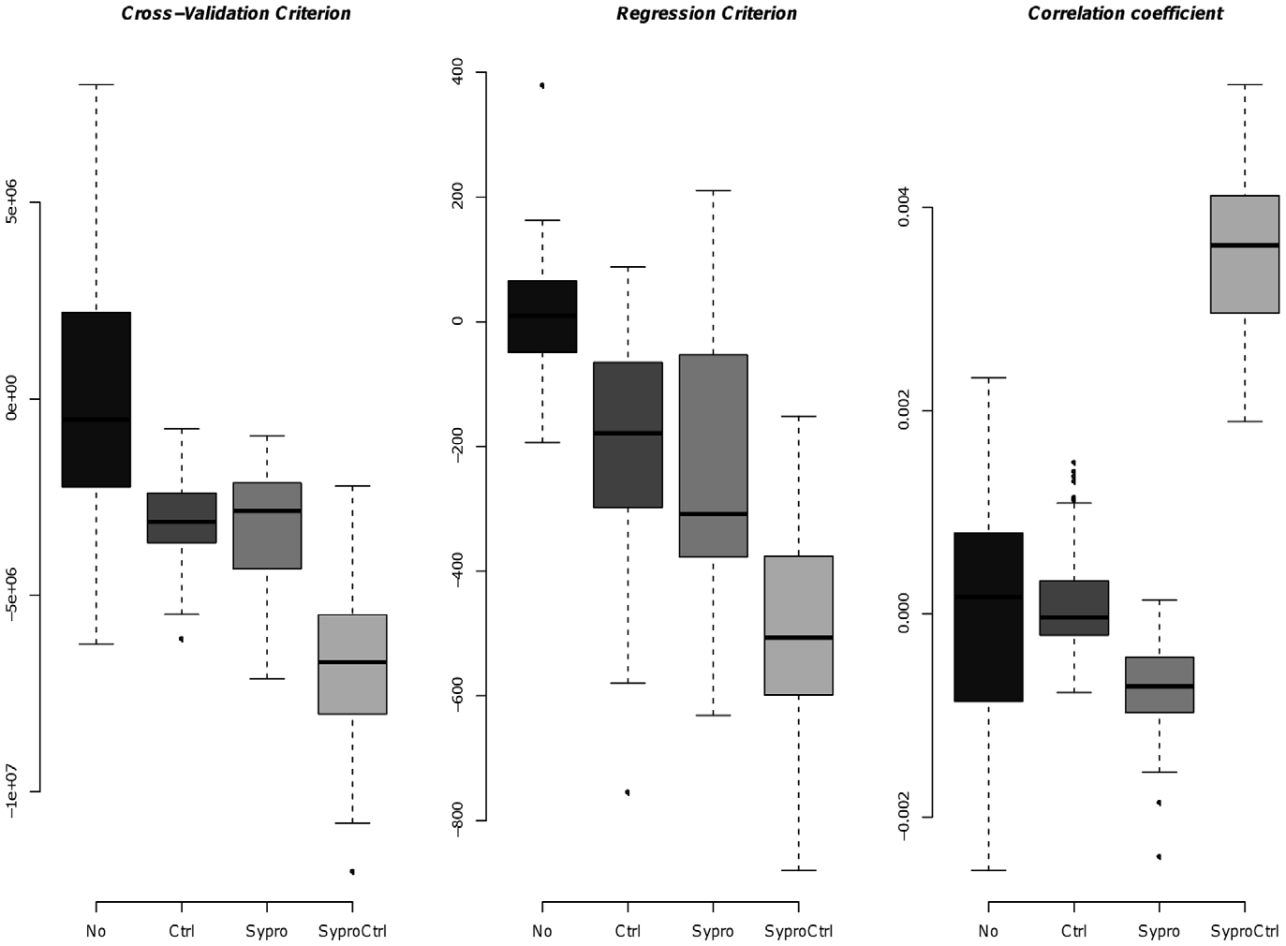
Prediction of protein expression: the benefit of using negative control and sypro ruby arrays. Summary of the three evaluation criteria (CV criterion, regression criterion and correlation coefficient) of the ModelSC1 for the five anti-chk2 arrays. No: No normalization (initial SuperCurve); Ctrl: addition of a negative control array; Sypro: addition of a total protein stained array; SyproCtrl: addition of both arrays. The initial SuperCurve (ModelSC1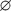) is used as a reference and set at 0 and the differences to this reference are computed for the other ModelSC1.

The results of the three validation criteria is shown in Figure 3. The ModelSC1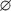 corresponds to the initial SuperCurve and is used as a reference (its criterion value is set at 0). Our models, including a negative control array (ModelSC1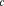), a sypro ruby array (ModelSC1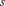) or both (ModelSC1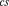), are compared to the initial SuperCurve model. The addition of one covariate (either ctrl or sypro) significantly improves the CV and regression criteria (but not the correlation coefficient) compared to the initial SuperCurve (p
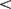
0.05). The results were validated by unilateral t-tests. Importantly, adding the two covariates (ctrl and sypro) even further improves the normalization, since all three criteria are significantly improved. Thus, the simultaneous quantification and normalization with the two covariates ctrl and sypro improves the robustness of the estimated protein expression levels. These results still hold if we consider ModelSC2, ModelSC3 and ModelSC4. Figure 3 summarizes the results obtained for the five anti-Chk2 arrays. The details of the criteria, array by array, can be found in Figure S2.

**Figure 4:**
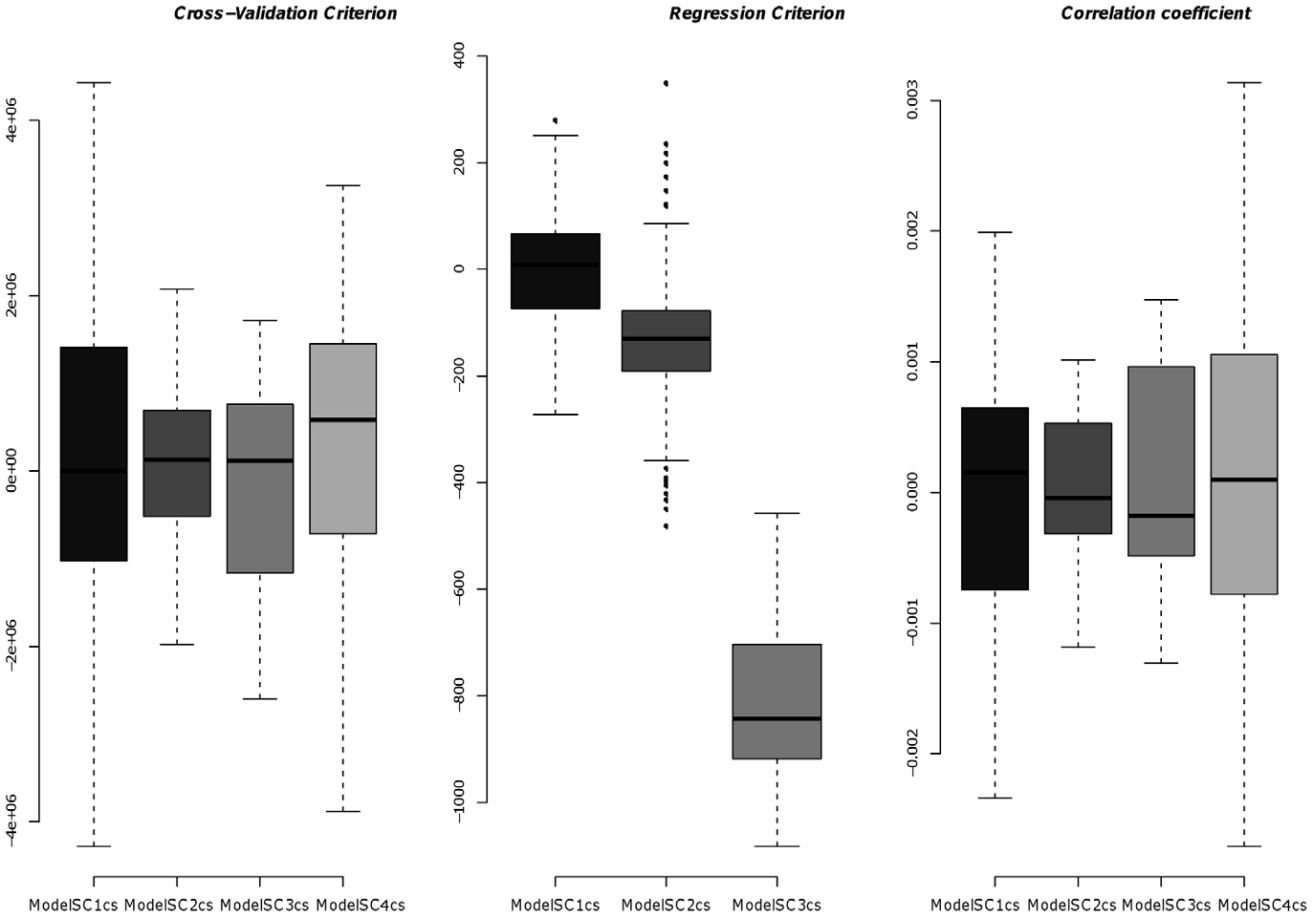
Prediction of protein expression: comparison between the models ModelSC1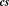, 2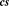, 3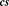 and 4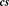. Summary of the three criteria (CV criterion, regression criterion and correlation coefficient) of the four ModelSC1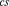, 2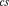, 3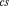 and 4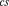, for the five anti-chk2 arrays. The ModelSC1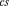 is used as a reference and set at 0. The differences to this reference are computed for the other ModelSC. The regression criterion cannot be calculated for the ModelSC4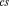, since this model includes a random effect.

Next, we compared the four models ModelSC1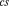, 2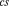, 3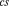 and 4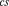, which all take into account a negative control array and a sypro ruby array, but differ in the effects that are taken into account (see Table 2). A summary of the results for the five anti-Chk2 arrays is given in the Figure 4 and validated by bilateral t-tests (the detailed array by array plots can be found in Figure S3). Figure 4 shows that the regression criterion is significantly lower in the model with the maximum of covariates (ModelSC3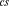). However, this result is not confirmed by the CV criterion or by the correlation coefficient, which are not significantly different across the four tested ModelSC. Thus, we conclude that, for the prediction of protein expression levels, the different models perform similarly, as long as they include both a ctrl and sypro array for normalization.

### Correction of the Total Amount of Spotted Proteins

The correction of RPPA data for the total amount of spotted protein is a crucial issue. Indeed, variations in total amounts of spotted protein are likely to happen involuntary in RPPA, mainly due to imprecise protein dosage methods. Without normalization, proteins could be erroneously considered as differentially expressed. In order to test the ability of our normalization models to correct for differences in the total amount of spotted protein, some samples were voluntarily spotted at varying amounts (0.8, 0.9, 1, 1.1 and 1.2 mg/ml starting concentrations). As expected, the sypro arrays permit to distinguish the total amounts of spotted proteins (Figure S4 ) and they may therefore be useful to remove this effect. Interestingly, ctrl arrays also reflect the spotted amounts (Figure S5 ). These arrays may therefore also be useful to normalize for variations in amounts of total protein.

Table 3 shows the p-values of the effect of the spotted amounts (0.8, 0.9, 1, 1.1 and 1.2 mg/ml), using the linear model 7 (see Material and Methods) applied on the ModelSC1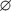, the ModelSC1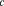, the ModelSC1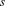, the ModelSC1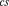. As expected, the ModelSC1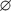 (*i.e* the initial SuperCurve model, without normalization) is not able to correct for the varying amounts: it estimates relative protein expression levels that are significantly different between the varying spotted amounts. This observation demonstrates the sensitivity of the RPPA technology and emphasizes the need for a normalization step. Indeed, such variations of 20% above or below the intended protein concentration are very likely to occur in RPPA. We here show that, without normalization, such variations significantly affect the estimated protein expression levels. Applying a model taking into account at least one covariate (ctrl and/or sypro) significantly improves the correction for the spotted amounts. In most cases, the p-value is no longer significant, indicating that the expression levels are considered similar after normalization. Similar results are obtained with the other ModelSC of Table 2.

**Table 3:** ModelSC 1: Capacity of the different models to normalize for the varying amounts of total protein spotted.

Array	Sample	pval φ	Pval*_s_*	pval*_c_*	pval*_cs_*
1	BSA	3.5969e-05	0.1449	0.0420	0.3053
2	BSA	8.2330e-06	0.0991	0.0802	0.2175
3	BSA	1.5165e-06	0.5173	0.0003	0.0063
4	BSA	3.9442e-04	0.3741	0.0016	0.2308
5	BSA	8.2330e-06	0.0991	0.0802	0.2175
1	3T3	3.3674e-08	0.0710	0.0616	0.0644
2	3T3	1.0865e-06	0.2752	0.0561	0.0536
3	3T3	2.1981e-07	0.1603	0.6549	0.2578
4	3T3	1.6289e-10	0.2555	0.1080	0.2672
5	3T3	1.0865e-06	0.2752	0.0561	0.0536

Represented are the p-values of the amount effects without neither 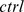 nor 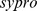 (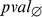), with 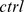 (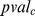), with 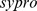 (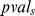), with 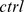 and 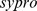 (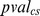).

In conclusion, the addition of at least one covariate (ctrl and/or sypro) significantly corrects the bias that could be induced by variations in the spotted amounts of total protein.

### Spatial Effects

Spatial bias are often found in micro-array technologies. They may be due to heterogeneous arrays or heterogeneous staining. Although the samples are spotted in a random manner onto the arrays, these spatial effects may lead to higher or lower signal in some rows and/or columns and thus bias the results. In this section, we will test if spatial effects are significantly present on raw data. Then, we test if the ModelSC of Table 2 are able to remove these effects.

For each ModelSC of Table 2, the significance of the effects 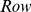 and 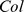 is studied via the Equation 8 in the Materials and Methods. By eye, we did not detect any spatial bias on the arrays. Yet, we detect for each array at least one significant spatial effect (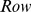 and/or 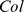 with p
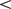
0.05) on the raw data. Only the ModelSC3, which takes into account the effects 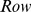 and 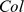 allows the total and reproducible removal of the spatial bias. For the ModelSC 1, 2 and 4, no trend can be found.

From the previous results, we can conclude that the best model is the ModelSC3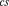, called NormaCurve from now on, which takes into account the ctrl and sypro arrays for normalization and the covariates 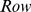 and 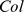 for correction of spatial bias.

### Reproducibility of Control Arrays

It has been shown that inter-RPPA comparison is challenging in RPPA due to high variability between slides [20]. Using a linear mixed-effect model (detailed in Methods S1), we indeed observe a significant difference in the raw data among the five replicate slides of Chk2, ctrl and sypro.

All results described before are performed with one ctrl array and one sypro array. Given the high inter-array variability, we hypothesized that using different ctrl and/or sypro arrays may lead to different results. Thus, all tests described above were reproduced with other combinations of ctrl and sypro arrays. Despite the high inter-array variability, a very good reproducibility of the results is obtained, since we confirm with all combinations of ctrl and sypro arrays that:

- Normalization with the two covariates ctrl and sypro improves the robustness of the estimated protein expression levels
- Only ModelSC3 completely removes the spatial effects.
- The addition of at least one covariate (ctrl and/or sypro) always leads to an improved normalization of the spotted amount of total protein.

To explain this reproducibility, we ranked all samples according to their estimated protein expression levels after normalization with different sets of ctrl and sypro arrays. A Wilcoxon test was then performed to compare the ranking of their estimated protein expression levels. The obtained p-values, close to 1, show that ranks of expression levels are conserved, no matter the used set of ctrl and sypro arrays. In conclusion, despite a strong inter-array variability, all ctrl and sypro arrays can be used for normalization without affecting reproducibility of the data.

### Required Number of Replicates

**Figure 5:**
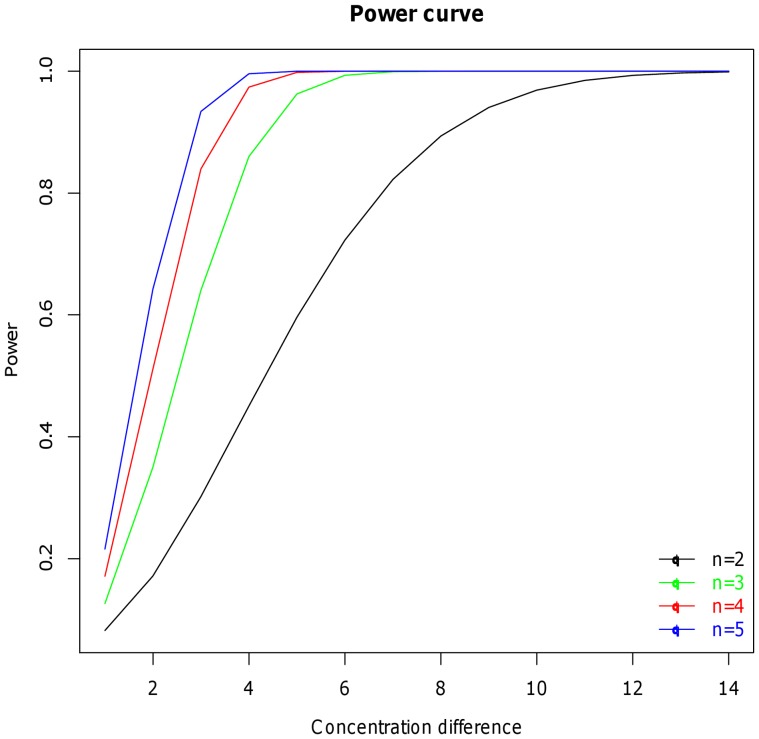
Power curves. Power curves showing the difference in relative protein expression that can be evidenced with 2, 3, 4 or 5 technical replicates.

Next, we address how many replicates are required per sample to evidence a given difference in expression level between two samples. For this, we first need to estimate the variability of the relative expression levels among replicates. Indeed, a higher variability among replicates means that more replicates will be required to significantly evidence a given difference in expression level. Using the linear model in Equation 9 (see Materials and Methods), we observe a significant inter-array variability, even on the normalized data. Figure 5 represents the power curves for increasing numbers of replicates (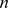 from 2 to 5) as a function of the difference in expression level (x-axis) varying from 2 to 15. The plot confirms that the higher the number of replicates, the faster the power grows, *i.e* the smaller the difference in expression needs to be. Moreover, not much power is gained between 3 to 5 replicates, meaning that convergence is close to be reached. In conclusion, with the variability observed in our experience, 3 replicates seems to be a good compromise between statistical power and space optimization on the arrays.

**Figure 6:**
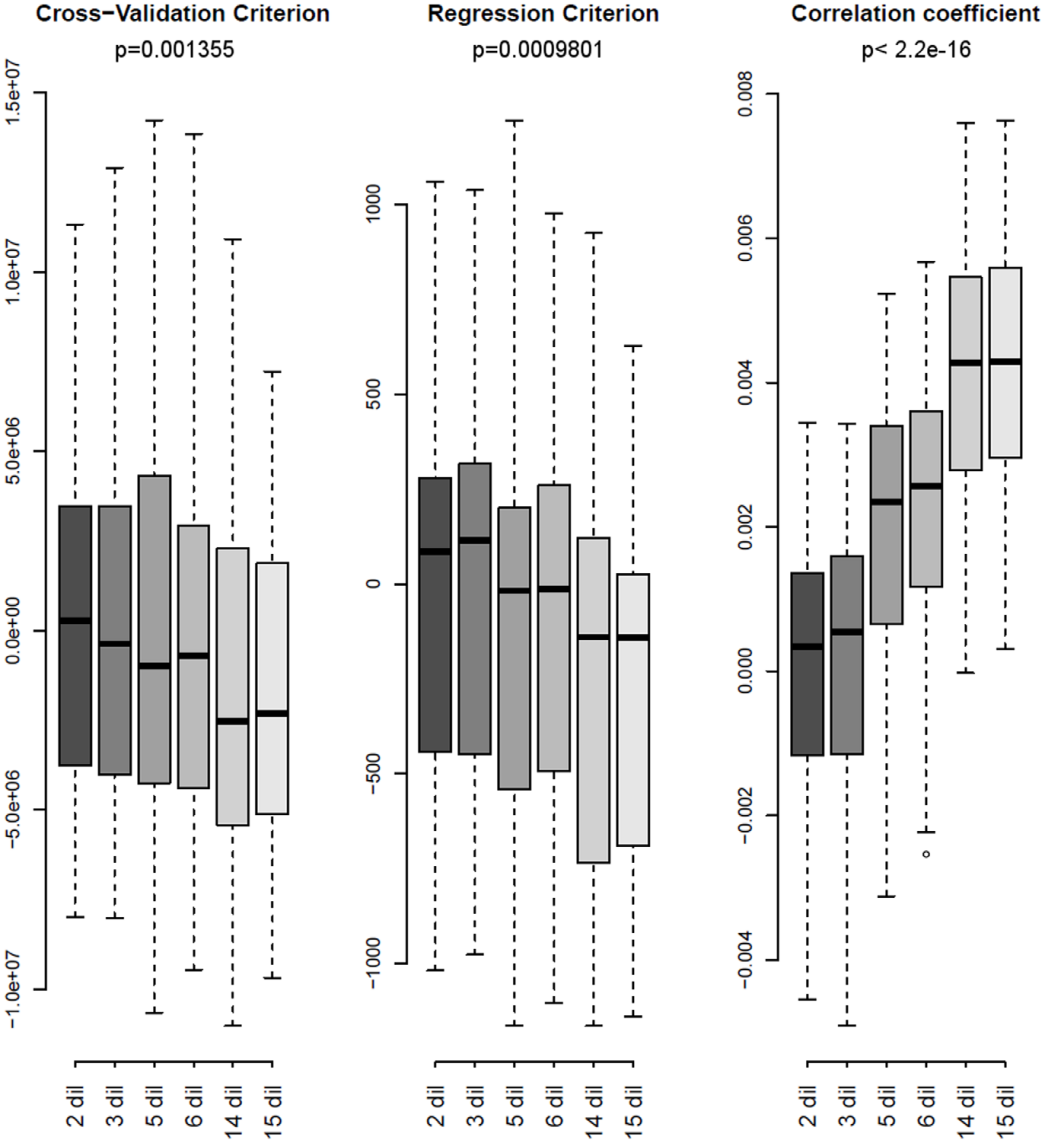
Protein expression prediction when using 2, 3, 5, 6, 14 or 15 serial dilutions for each sample. Summary of the three evaluation criteria (CV criterion, regression criterion and correlation coefficient) for the five anti-chk2 arrays. The model using 2 dilutions is used as a reference and set at 0. The differences to this reference are computed for the other number of dilutions. p-values (Anova test) are indicated.

### Required Number of Serial Dilutions

In our experiment, 15 serial dilutions were printed for each sample, allowing robust curve fitting and thus optimal estimation of relative protein expression. However, the highest dilution steps show intensities at background levels and may thus not be essential for the curve fitting. In addition, when many samples need to be analyzed on the same array, the number of serial dilutions needs to be reduced due to space limitation. Therefore, we studied how many serial dilutions are required for robust estimation of expression. For this, we took into account only the 2, 3, 5, 6 or 14 less diluted (most concentrated) dilution steps of each sample and compared this to all 15 dilutions. Relative expression levels were estimated with these varying numbers of dilutions. We then compared estimated expression levels with true protein concentrations for the two most concentrated dilutions steps, which were the two points in common among the different analyses. We computed the regression criterion and the correlation coefficient as in Figure 3 while for the cross-validation criterion, only the two common points were considered. We observe that all three criteria significantly improve when the number of dilutions increases. Moreover, a distinct improvement occurs between 3 and 5 dilutions, notably for the correlation coefficient and the regression criterion. We therefore conclude that 5 serial dilutions per sample is a good compromise between robust estimation of expression levels and space optimization on the arrays.

## Discussion

In this article, we propose a method to simultaneously quantify and normalize RPPA data, based on the initial quantification algorithm SuperCurve [15]. We show that the best normalization model, which we call NormaCurve, takes into account the two control arrays ctrl and sypro, as well as the two spatial effects 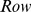 and 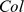. This model, validated by cross-validation, allows the correction of spatial effects and corrects for differences in total amount of spotted proteins. When we compared NormaCurve with the initial SuperCurve model, significantly better correlations with the true concentrations were obtained with NormaCurve. Notably, we addressed the crucial issue of varying protein amounts. We show that, in contrast to the initial SuperCurve model, NormaCurve is able to correct for the total amount of spotted protein when this latter varies between 1.2 and 0.8 mg/ml for the first dilution step. Within this range, involuntary variation in the concentration of a sample will thus be corrected by NormaCurve and will not bias obtained results. A next step will be to study up to which variability in the deposited amounts of protein the normalization is satisfactory.

Interestingly, to correct for the total amount of spotted protein, taking into account the two control arrays (both ctrl and sypro) is not always better than taking either ctrl or sypro (Table 3). This suggests that these two slides partially vehicle the same information. Indeed, we observed that the background fluorescence measured on the ctrl slide correlates with the total amount of spotted protein. However, the ctrl array has an antibody-based detection, similar to the protein detection protocol, while the sypro array is a chemical staining procedure. The two slides are therefore not expected to be entirely overlapping. Indeed, we observe that inclusion of both ctrl and sypro arrays in our model estimates relative protein expression levels that better reflect true protein concentrations, compared to either ctrl or sypro alone (Figure 3). Thus, ctrl and sypro arrays are complementary and should both be included for an optimal normalization of the data.

The advantage of Normacurve, compared to microenvironment normalization [21], is that control lysates are not required for normalization. Thus, the entire array could be used for samples of interest. NormaCurve is also attractive compared to median-based normalization, in which the median of all arrays is simply set to the same level for each sample. Indeed, in contrast to median-based normalization, NormaCurve does not require a minimum amount of antibodies to be powerful and it is not affected by a bias in the chosen antibodies. Thus, NormaCurve appears as the most versatile and useful normalization method currently available for RPPA data.

In our experiment, we observed a significant difference among replicate arrays, thus confirming the high inter-array variability described elsewhere [20]. This implies that, when an important number of samples are to be analyzed, these might better not be divided over several arrays. Rather, a decreased number of technical replicates and/or of dilution steps should be used for each sample. Diminishing the number of serial dilutions impairs the reliability of the estimated expression levels (Figure 6), while diminishing the number of technical replicates affects the power in the subsequent statistical analysis (Figure 5). We show that the optimal compromise between data robustness and space optimization on the arrays consists of printing each sample in 5 serial dilutions and 3 technical replicates.

All dilution steps, replicates and samples should be distributed as randomly as possible over the array. This will make it possible to optimize the normalization of potential spatial bias and ensure an efficient identification of the relevant biological differences between the samples under investigation.

In RPPA, spatial bias is mainly due to intrinsic heterogeneity of nitrocellulose on the slides and to heterogeneous staining. NormaCurve proposes a spatial normalization via two linear covariates (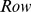 and 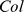). Such spatial bias is a recurrent problem in the microarray field and different methods have been proposed to correct this artifact [24], [25]. The MANOR method initially developed for array-CGH experiment [24] did not perform better than the proposed model (not shown). This may be explained by the number of spots on RPPA arrays, which is too low to efficiently estimate a spatial trend. However, future technical improvements, such as diminished spot size through the use of smaller spotting pins, might allow increasing the density of spots on the arrays. Spatial normalization method would then deserve to be re-evaluated.

## Supporting Information

Figure S1 For a Chk2-labeled array, mean intensities were plotted against the Coefficient of Variation for all samples and all dilution steps. Note that high CVs are associated with low intensities.
(TIF)

Figure S2 CV criterion, regression criterion and correlation coefficient of the ModelSC 1 for the five arrays stained with anti-Chk2.
(TIF)

Figure S3Comparison of the ModelSC1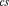, 2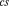, 3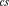 and 4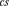 for the five arrays stained with anti-Chk2.(TIF)

Figure S4 Observed intensities on a Sypro Ruby stained array for the dilution series of the BSA+chk2 samples with five different starting concentrations (0.8, 0.9, 1, 1.1 and 1.2 mg/ml). The sypro array correctly distinguishes between the different starting concentrations.
(TIF)

Figure S5 Observed intensities on a control array (no primary antibody) for the dilution series of the BSA+chk2 samples with five different starting concentrations (0.8, 0.9, 1, 1.1 and 1.2 mg/ml). The ctrl array distinguishes between the different starting concentrations.
(TIF)

Methods S1 Reproductibility of control arrays.
(PDF)
