# Supplementary figures and images for: NormaCurve: A SuperCurve-Based Method That Simultaneously Quantifies and Normalizes Reverse Phase Protein Array Data
Source: PLoS One. 2012 Jun 28;7(6):e38686. doi: 10.1371/journal.pone.0038686 (PMC3386279; doi:10.1371/journal.pone.0038686)

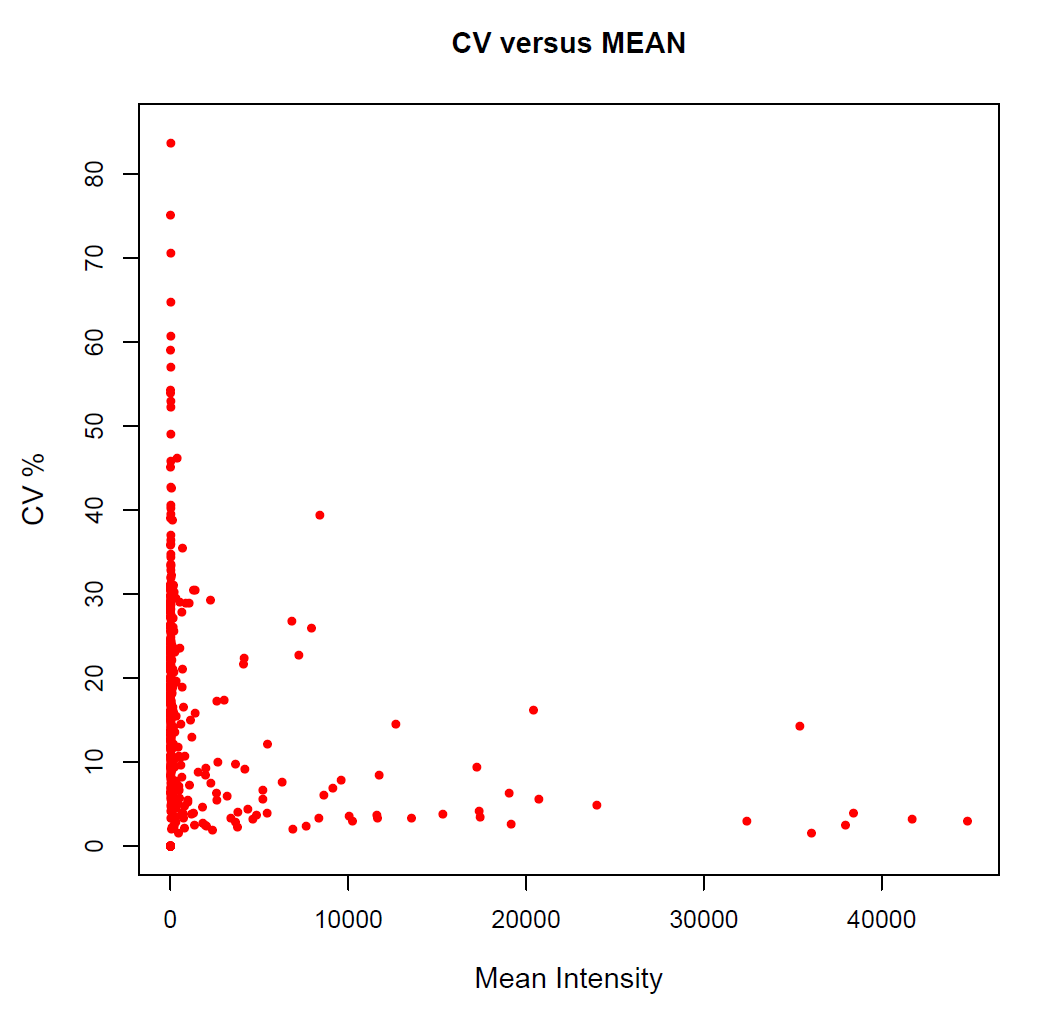

Supplement: Figure S1 — For a Chk2-labeled array, mean intensities were plotted against the Coefficient of Variation for all samples and all dilution steps. Note that high CVs are associated with low intensities. (TIF) [file pone.0038686.s001.tif]

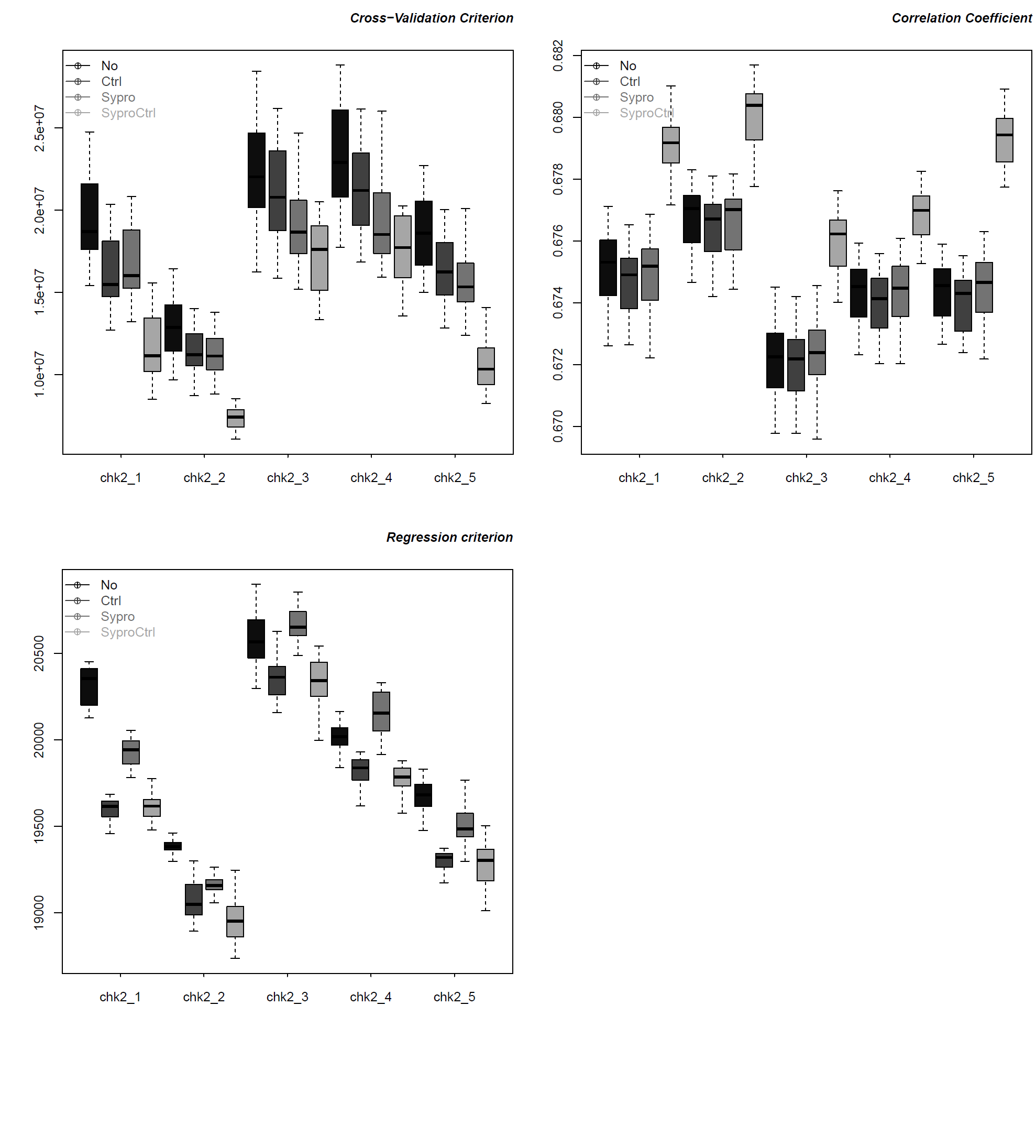

Supplement: Figure S2 — CV criterion, regression criterion and correlation coefficient of the ModelSC 1 for the five arrays stained with anti-Chk2. (TIF) [file pone.0038686.s002.tif]

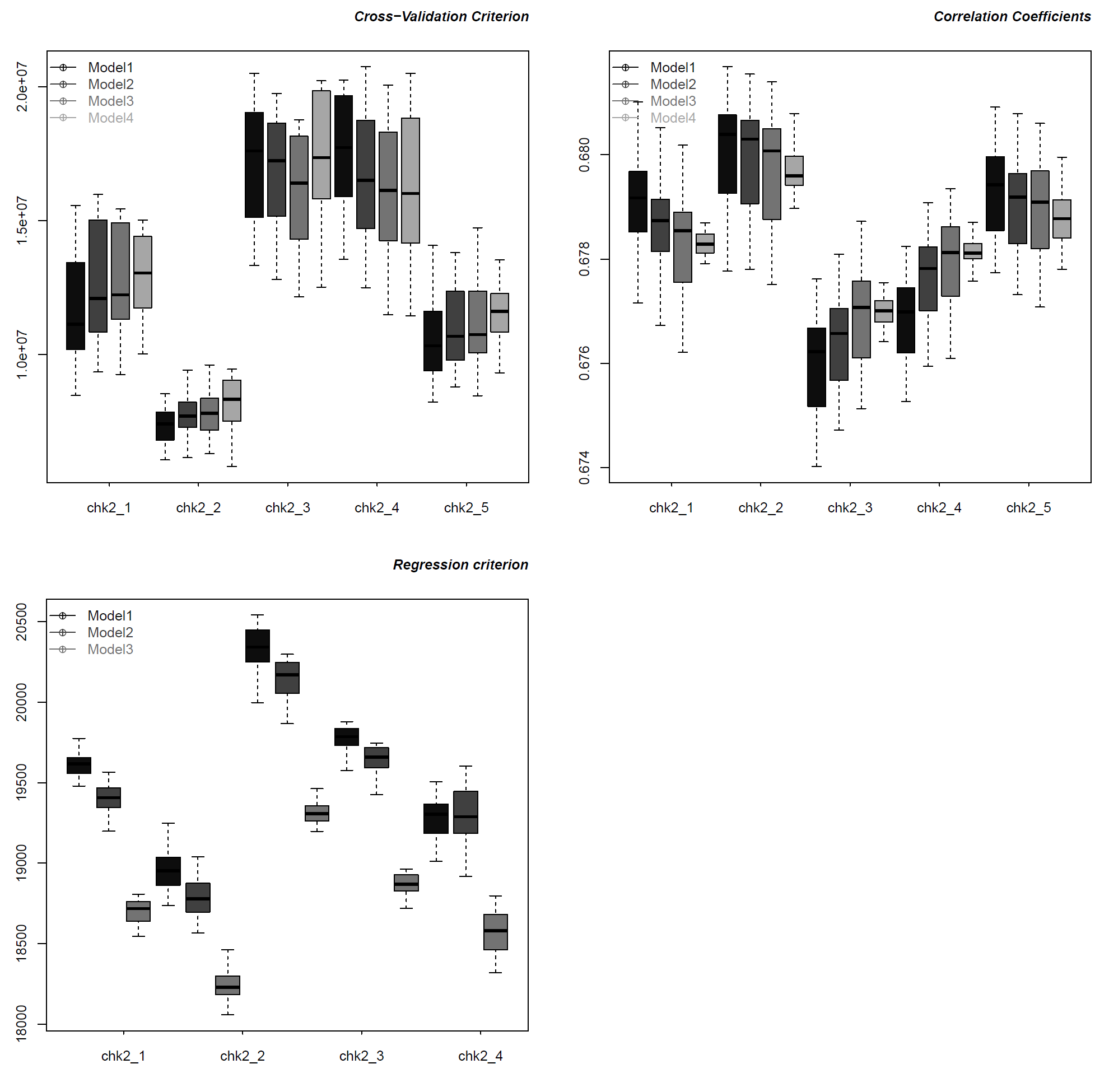

Supplement: Figure S3 — Comparison of the ModelSC1, 2, 3 and 4 for the five arrays stained with anti-Chk2. (TIF) [file pone.0038686.s003.tif]

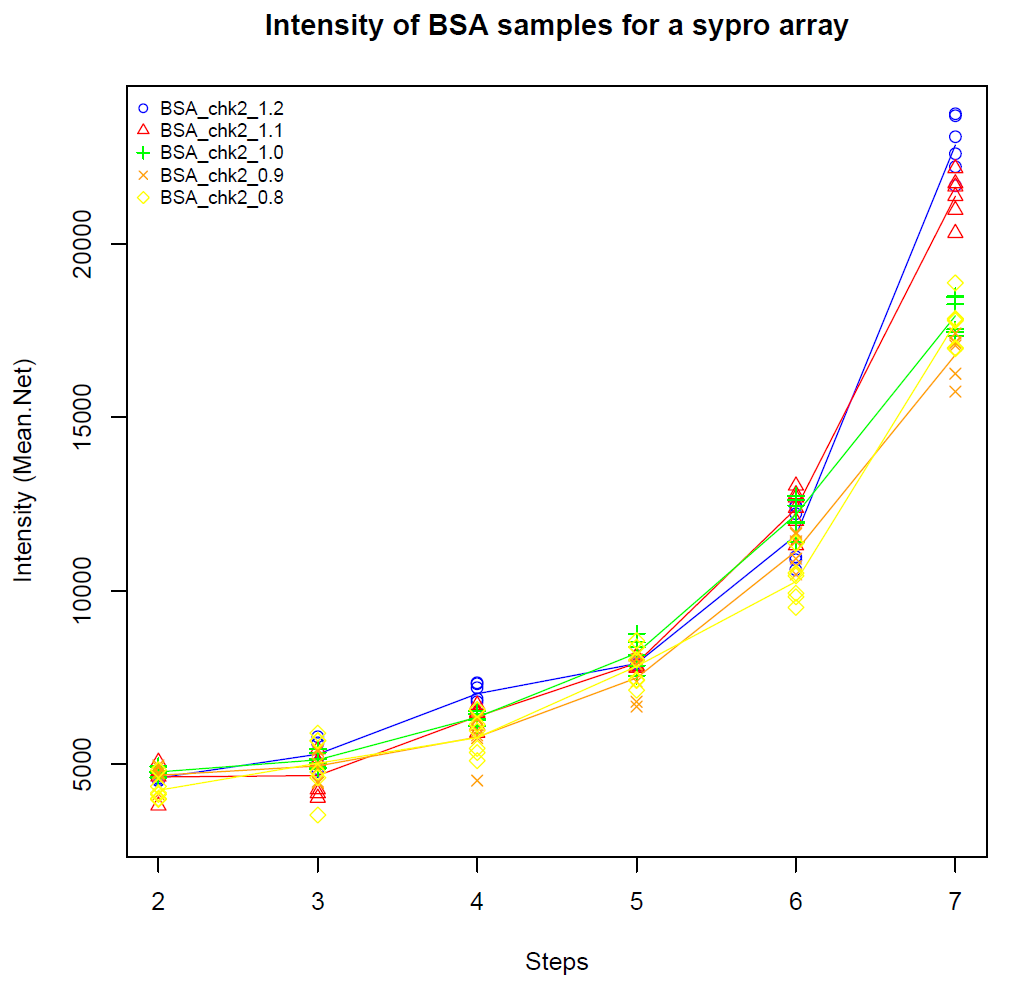

Supplement: Figure S4 — Observed intensities on a Sypro Ruby stained array for the dilution series of the BSA+chk2 samples with five different starting concentrations (0.8, 0.9, 1, 1.1 and 1.2 mg/ml). The sypro array correctly distinguishes between the different starting concentrations. (TIF) [file pone.0038686.s004.tif]

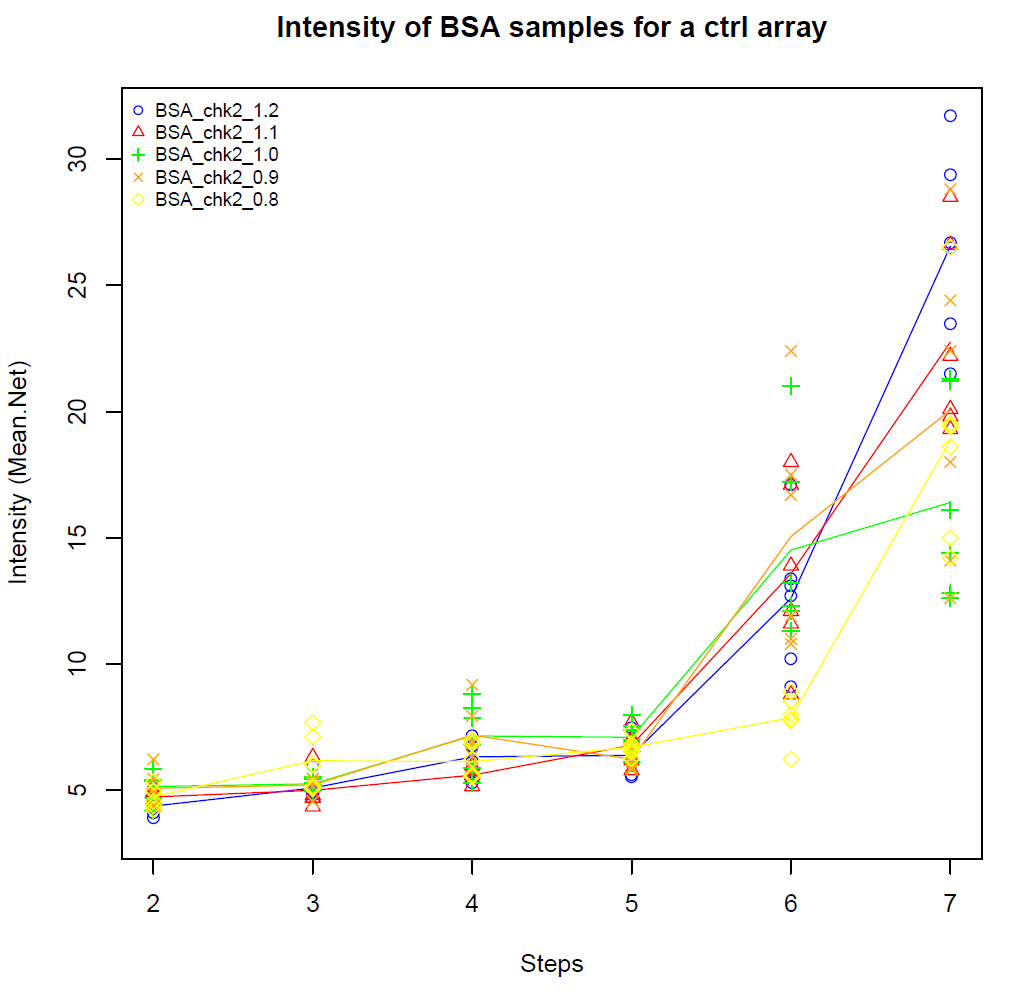

Supplement: Figure S5 — Observed intensities on a control array (no primary antibody) for the dilution series of the BSA+chk2 samples with five different starting concentrations (0.8, 0.9, 1, 1.1 and 1.2 mg/ml). The ctrl array distinguishes between the different starting concentrations. (TIF) [file pone.0038686.s005.tif]
