# Supplementary material for: NormaCurve: A SuperCurve-Based Method That Simultaneously Quantifies and Normalizes Reverse Phase Protein Array Data
Source: PLoS One. 2012 Jun 28;7(6):e38686. doi: 10.1371/journal.pone.0038686 (PMC3386279; doi:10.1371/journal.pone.0038686)
Supplement: Methods S1 — Reproductibility of control arrays. (PDF) [file pone.0038686.s006.pdf]

Reproductibility of control arrays

In order to assess the inter-RPPA variability in the raw data for the three kinds of array (ctrl, sypro and anti-chk2), the following linear mixed-effect model was used:

$$y_{ijkl} = \mu + Sample_i + Steps_j + Array_k + \epsilon_{ijkl} \quad (1)$$

where:

- y_{ijkl} corresponds to the raw intensity,
- μ corresponds to the overall mean,
- $Sample_i$ corresponds to the sample fixed-effect,
- $Steps_j$ corresponds to the dilution step fixed-effect,
- $Array_k$ corresponds to the array random-effect, with $Array_k \sim \mathcal{N}(0, \sigma_{array})$ and σ_{array} corresponds to the inter-RPPA variability,
- ϵ_{ijkl} corresponds to the residual error with $\epsilon_{ijkl} \sim \mathcal{N}(0, \sigma_r)$ and σ_r corresponds to the intra-RPPA variability.

We tested if the inter-array variability is significant relative to the intra-RPPA variability. No matter the type of array, the null hypothesis is significantly rejected. This demonstrates that in the raw data, the inter-array variability is significantly higher than the intra-array variability.
